# Pretreatment cardiometabolic risk in newly diagnosed breast cancer: a molecular subtype analysis

**DOI:** 10.3389/fcvm.2026.1835915

**Published:** 2026-06-10

**Authors:** Zechang Xin, Xiaoyu Zhu, Hui Qu, Xing Ai, Kaixuan Wang, Xinyue Li, Ming Wang, Pisong Li, Zhongbin Han, Ningxin Qu, Weiting Yu, Hongshen Chen

**Affiliations:** 1 Department of Breast and Thyroid Surgery, Affiliated Zhongshan Hospital of Dalian University, Dalian, China; 2 Central Laboratory, Affiliated Zhongshan Hospital of Dalian University, Dalian, China

**Keywords:** breast cancer, cardio-oncology, cardiovascular comorbidity, dyslipidemia, molecular subtype

## Abstract

**Background:**

Breast cancer is the most common female malignancy globally. With improved survival, cardiovascular diseases have become a key morbidity source due to shared cardiometabolic risk factors (e.g., dyslipidemia). While cardiovascular risk varies by breast cancer molecular subtype, baseline pre-treatment cardiovascular comorbidities and lipid abnormalities remain undercharacterized. We aimed to describe their prevalence and subtype-specific patterns in newly diagnosed patients.

**Methods:**

This single-center retrospective cohort study included 5,246 Chinese women with newly diagnosed breast cancer (2014–2024), with all data collected before anticancer therapy. Patients were stratified into five molecular subtypes. Baseline cardiovascular comorbidities, tumor features, and serum lipids were analyzed using chi-square tests, ANOVA, and multivariable logistic regression.

**Results:**

Age, cardiovascular comorbidities, electrocardiographic abnormalities and lipid profiles differed significantly across subtypes. Overall dyslipidemia prevalence was 54.1%, highest in Luminal A and lowest in Luminal B (HER2 negative). Intravascular tumor thrombus was independently associated with dyslipidemia in all subtypes, with the strongest association in triple-negative breast cancer. Lymph node metastasis correlated with dyslipidemia only in Luminal B subtypes, and several factors showed subtype-specific associations.

**Conclusions:**

Dyslipidemia is common at baseline in treatment-naive breast cancer patients and varies by molecular subtype. Tumor-related features, particularly intravascular tumor thrombus, are consistently associated with dyslipidemia. Early lipid screening and cardiovascular risk assessment may optimize cardio-oncology care.

## Background

Breast cancer is the most frequently diagnosed malignancy among women worldwide. With advances in screening, pathology-guided classification, and multimodal treatment strategies, survival has improved substantially (1–3). As longevity increases, non-cancer comorbidities have become increasingly important determinants of overall health and long-term outcomes (4). Among these, cardiovascular diseases represent a major source of morbidity in patients with breast cancer (5). Cardiometabolic risk factors such as dyslipidemia, hypertension, and diabetes mellitus are common in the general population and may coexist at the time of cancer diagnosis (6–9). In addition to shared epidemiologic risk factors, cancer-related systemic inflammation and tumor–host interactions may influence lipid metabolism and vascular homeostasis (7). Subsequent anticancer therapies can further modify cardiometabolic risk and contribute to cardiovascular morbidity (10).

Breast cancer is biologically heterogeneous. Molecular subtypes defined by hormone receptor and HER2 status differ in tumor biology, clinical course, and treatment paradigms, which may translate into distinct baseline cardiometabolic profiles (11, 12). However, evidence describing baseline cardiovascular comorbidities and lipid abnormalities at the time of first diagnosis—before initiation of any anticancer therapy—remains limited, particularly in analyses stratified by molecular subtype (13). Establishing pretreatment cardiovascular and lipid profiles may support risk stratification at diagnosis and provide a reference for interpreting cardiometabolic changes during subsequent therapy and follow-up.

Beyond treatment-related cardiotoxicity, pre-existing cardiometabolic disorders at diagnosis may influence cardiovascular reserve and complicate subsequent cancer management (14, 15). Cancer-related systemic inflammation, endocrine alterations, and tumor–host interactions can disrupt lipid handling and vascular homeostasis, potentially contributing to dyslipidemia and early cardiovascular vulnerability even before anticancer therapy is initiated (16–18).Therefore, defining pretreatment cardiometabolic profiles provides an essential reference for disentangling baseline risk from therapy-associated metabolic changes during follow-up (19, 20).

Current evidence on cardiovascular comorbidities and lipid abnormalities in breast cancer is largely derived from treatment-exposed cohorts or long-term survivors, where baseline laboratory measurements and subtype-stratified clinicopathological variables may be incomplete (13, 21). Moreover, heterogeneity in dyslipidemia definitions and lipid thresholds limits comparability across studies and hampers clinical translation (22, 23). Real-world data describing pretreatment cardiovascular comorbidities, electrocardiographic findings, and dyslipidemia phenotypes across molecular subtypes at first diagnosis remain limited, particularly in routine clinical settings (24, 25).

Therefore, this study aimed to (i) characterize baseline cardiovascular comorbidities, electrocardiographic abnormalities, and lipid abnormalities in newly diagnosed, treatment-naive breast cancer patients; (ii) compare the prevalence and patterns of dyslipidemia across molecular subtypes; and (iii) explore subtype-specific factors associated with dyslipidemia using multivariable regression models.

## Methods

### Study design and population

**Figure 1:**
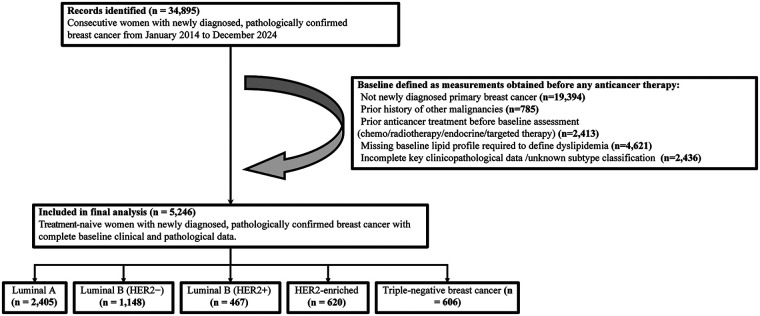
Study flowchart. Selection of 5,246 treatment-naive women with newly diagnosed.

This single-center retrospective cohort study was conducted at the Affiliated Zhongshan Hospital of Dalian University (Dalian, China). We reviewed consecutive female patients with newly diagnosed, pathologically confirmed breast cancer between January 2014 and December 2024. Only baseline information collected at diagnosis and before initiation of any anticancer treatment including surgery, chemotherapy, radiotherapy, endocrine therapy, or targeted therapy was analyzed (Figure 1).

Eligible patients were adults with primary breast cancer confirmed by pathology and available baseline clinical data and serum lipid measurements. Patients were excluded if they had received any anticancer treatment prior to baseline assessment, had a history of another malignancy, or lacked key lipid measurements required to define dyslipidemia. The study protocol was approved by the Institutional Review Board of the Affiliated Zhongshan Hospital of Dalian University (Approval No.: PJ-KY2026-016-1). Owing to the retrospective nature and use of de-identified data, the requirement for written informed consent was waived in accordance with institutional policies.

### Molecular subtype classification

Molecular subtypes were defined based on estrogen receptor (ER), progesterone receptor (PR), and human epidermal growth factor receptor 2 (HER2) status according to standard clinical breast cancer classification. Luminal A was defined as ER positive, PR positive, and HER2 negative. Luminal B (HER2 negative) was defined as ER positive, PR negative, and HER2 negative. Luminal B (HER2 positive) was defined as ER positive, any PR status, and HER2 positive. HER2-enriched was defined as ER negative, PR negative, and HER2 positive. Triple-negative breast cancer (TNBC) was defined as ER negative, PR negative, and HER2 negative. All subtype classifications were obtained from final pathological diagnosis reports. The final cohort comprised Luminal A (*n* = 2,405), Luminal B (HER2 negative) (*n* = 1,148), Luminal B (HER2 positive) (*n* = 467), HER2-enriched (*n* = 620), and TNBC (*n* = 606).

### Data collection and variables

Baseline variables were extracted from electronic medical records, including demographics, medical history, electrocardiographic findings, tumor pathology and staging indicators, laboratory indices, and tumor markers. Cardiovascular comorbidities included clinician-diagnosed hypertension, diabetes mellitus, coronary heart disease (CHD) and electrocardiographic abnormalities. Electrocardiographic abnormalities were recorded from Electrocardiogram (ECG) reports and clinical documentation, including any arrhythmia, such as sinus bradycardia, sinus tachycardia, sinus arrhythmia, atrial fibrillation, and atrioventricular block.

Baseline serum lipid profiles included low-density lipoprotein cholesterol (LDL-C), high-density lipoprotein cholesterol (HDL-C), total cholesterol (TC), and triglycerides (TG). Dyslipidemia and lipid abnormality patterns were defined according to the dataset used in this study and summarized as counts and subtype-specific prevalences. Tumor-related variables included lymph node metastasis, intravascular tumor thrombus, clinical stage, and TNM components, where T represents the size and tumor invasion depth of the primary tumor, N indicates regional lymph node involvement, and M represents distant metastasis. Molecular pathology indicators E-cadherin, TOPOII, P120 catenin, and CK5/6 were independently evaluated by two senior board-certified pathologists blinded to clinical and laboratory data. Any discrepancies were resolved by consensus review. Intravascular tumor thrombus was defined as the presence of tumor emboli within vascular lumens on routine H&E-stained sections. All immunohistochemical markers were assessed according to standardized institutional pathological criteria and scoring protocols, applied consistently throughout the study period. Baseline laboratory indices (estradiol, follicle-stimulating hormone, serum creatinine, uric acid, body mass index) and tumor markers (CEA, carcinoembryonic antigen; CA125, cancer antigen 125; CA153, cancer antigen 153) were also extracted from clinical and pathological records.

### Lipid control categorization

Lipid parameters were categorized according to the Chinese guidelines for the management of dyslipidemia in adults (26): The cut-off values were defined as follows: LDL-C: <2.6, 2.6–<3.4, 3.4–<4.1, and ≥4.1 mmol/L; HDL-C: <1.0 mmol/L; non-high-density lipoprotein cholesterol (non-HDL-C): <3.4, 3.4–<4.1, 4.1–<4.9, and ≥4.9 mmol/L; total cholesterol (TC): <5.2, 5.2–<6.2, and ≥6.2 mmol/L; and triglycerides (TG): <1.7, 1.7–<2.3, and ≥2.3 mmol/L. Non-HDL-C was calculated as TC minus HDL-C. Dyslipidemia was defined as the presence of at least one abnormal lipid parameter, including elevated TC (≥5.2 mmol/L), elevated non-HDL-C (≥3.4 mmol/L), low HDL-C (<1.0 mmol/L), or LDL-C (≥ 4:1 mmol/L) consistent with the above clinical practice guidelines.

### Statistical analysis

Continuous variables are presented as mean ± standard deviation, and categorical variables as counts (percentages). Differences across molecular subtypes were assessed using one-way ANOVA for continuous variables and chi-square tests for categorical variables. Subtype-stratified multivariable logistic regression models were constructed to identify factors associated with dyslipidemia within each subtype; results are reported as odds ratios (ORs) with 95% confidence intervals (CIs). A two-sided *p*-value < 0.05 was considered statistically significant. All analyses were performed using SPSS version 25.0 (IBM Corp., Armonk, NY, USA).

## Results

### Baseline characteristics

A total of 5,246 treatment-naive patients with newly diagnosed breast cancer were included (Table 1). The mean age of the cohort was 55.41 ± 9.30 years and differed significantly across molecular subtypes (*p* < 0.001), with Luminal A patients being oldest on average (56.62 ± 8.25 years) and Luminal B (HER2−) patients youngest (52.74 ± 10.92 years). Baseline hypertension was documented in 306 patients (5.8%) and was most frequent in Luminal B (HER2−) (172/1,148; 15.0%) and least frequent in Luminal A (37/2,405; 1.5%) (*p* < 0.001). Diabetes mellitus was present in 124 patients (2.4%), with the highest prevalence in Luminal B (HER2−) (69/1,148; 6.0%) (*p* < 0.001). CHD was recorded in 246 patients (4.7%) overall and was most common in Luminal B (HER2−) (134/1,148; 11.7%) (*p* < 0.001). Electrocardiographic abnormalities differed by subtype. Any arrhythmia was uncommon (53 patients; 1.0%) but varied across subtypes (*p* < 0.001). Tumor-related indicators demonstrated heterogeneity: lymph node metastasis was present in 3,553 patients (67.7%) overall (*p* = 0.003), and intravascular tumor thrombus was present in 3,059 (58.3%) overall (*p* < 0.001). Clinical stage distributions differed across subtypes (all *p* < 0.001).

**Table 1:** Baseline characteristics of treatment-naive patients with newly diagnosed breast cancer by molecular subtype.

Characteristic	Luminal A (*n* = 2,405)	Luminal B (HER2⁻) (*n* = 1,148)	Luminal B (HER2⁺) (*n* = 467)	HER2-enriched (*n* = 620)	Triple negative (*n* = 606)	Total (*n* = 5,246)	*P* value
Age	56.62 ± 8.25	52.74 ± 10.92	54.46 ± 9.29	56.21 ± 8.14	55.55 ± 9.9	55.41 ± 9.3	<0.001
Hypertension	37 (1.50%)	172 (15.00%)	31 (6.60%)	36 (5.80%)	30 (5.00%)	306 (5.80%)	<0.001
Diabetes mellitus	21 (0.90%)	69 (6.00%)	6 (1.30%)	16 (2.60%)	12 (2.00%)	124 (2.40%)	<0.001
CHD	34 (1.40%)	134 (11.70%)	22 (4.70%)	31 (5.00%)	25 (4.10%)	246 (4.70%)	<0.001
Electrocardiographic Abnormalities							
Any arrhythmia	4 (0.20%)	35 (3.00%)	3 (0.60%)	4 (0.60%)	7 (1.20%)	53 (1.00%)	<0.001
Sinus bradycardia	8 (0.30%)	46 (4.00%)	8 (1.70%)	10 (1.60%)	13 (2.10%)	85 (1.60%)	<0.001
Sinus tachycardia	1 (0.00%)	10 (0.90%)	1 (0.20%)	0 (0.00%)	2 (0.30%)	14 (0.30%)	<0.001
Sinus arrhythmia	1 (0.00%)	8 (0.70%)	2 (0.40%)	1 (0.20%)	1 (0.20%)	13 (0.20%)	<0.001
Atrial fibrillation	0 (0.00%)	4 (0.30%)	0 (0.00%)	4 (0.60%)	2 (0.30%)	10 (0.20%)	0.006
Atrioventricular block	2 (0.10%)	15 (1.30%)	0 (0.00%)	0 (0.00%)	2 (0.30%)	19 (0.40%)	<0.001
Tumor pathology & staging indicators							
Lymph node metastasis	1,588 (66.00%)	765 (66.60%)	332 (71.10%)	457 (73.70%)	411 (67.80%)	3,553 (67.70%)	0.003
Intravascular tumor thrombus	1,371 (57.00%)	393 (34.20%)	294 (63.00%)	502 (81.00%)	499 (82.30%)	3,059 (58.30%)	<0.001
Clinical stage							<0.001
1	1,774 (73.80%)	715 (62.30%)	292 (62.50%)	396 (63.90%)	391 (64.50%)	3,568 (68.00%)	
2	252 (10.50%)	184 (16.00%)	93 (19.90%)	106 (17.10%)	100 (16.50%)	735 (14.00%)	
3	343 (14.30%)	227 (19.80%)	73 (15.60%)	104 (16.80%)	107 (17.70%)	854 (16.30%)	
4	36 (1.50%)	22 (1.90%)	9 (1.90%)	14 (2.30%)	8 (1.30%)	89 (1.70%)	
T							<0.001
1	862 (35.80%)	421 (36.70%)	163 (34.90%)	208 (33.50%)	197 (32.50%)	1,851 (35.30%)	
2	1,036 (43.10%)	504 (43.90%)	204 (43.70%)	260 (41.90%)	255 (42.10%)	2,259 (43.10%)	
3	272 (11.30%)	100 (8.70%)	57 (12.20%)	88 (14.20%)	83 (13.70%)	600 (11.40%)	
4	235 (9.80%)	123 (10.70%)	43 (9.20%)	64 (10.30%)	71 (11.70%)	536 (10.20%)	
N							<0.001
1	987 (41.00%)	440 (38.30%)	189 (40.50%)	267 (43.10%)	251 (41.40%)	2,134 (40.70%)	
2	345 (14.30%)	167 (14.50%)	92 (19.70%)	108 (17.40%)	88 (14.50%)	800 (15.20%)	
3	256 (10.60%)	158 (13.80%)	51 (10.90%)	82 (13.20%)	72 (11.90%)	619 (11.80%)	
M							
1	36 (1.50%)	22 (1.90%)	9 (1.90%)	14 (2.30%)	8 (1.30%)	89 (1.70%)	0.602
Molecular pathology indicators							
E-cadherin	57 (2.40%)	500 (43.60%)	94 (20.10%)	86 (13.90%)	153 (25.30%)	890 (17.00%)	<0.001
TOPOII	1,028 (42.70%)	794 (69.20%)	371 (79.40%)	116 (18.70%)	200 (33.10%)	2,509 (47.80%)	<0.001
P120 catenin	630 (26.20%)	442 (38.50%)	174 (37.30%)	550 (88.70%)	246 (40.70%)	2,042 (38.90%)	<0.001
CK5/6	195 (8.10%)	25 (2.20%)	294 (63.00%)	484 (78.10%)	355 (58.70%)	1,353 (25.80%)	<0.001
Biochemical & lipid indicators							
Dyslipidemia	1,424 (59.2%)	504 (43.9%)	254 (54.4%)	341 (55.0%)	315 (52.0%)	2,838 (54.1%)	<0.001
LDL (mmol/L)	3.1 ± 1.66	2.62 ± 0.97	2.89 ± 1.53	2.94 ± 1.59	3.01 ± 1.47	2.95 ± 1.51	<0.001
HDL (mmol/L)	1.11 ± 0.40	1.22 ± 0.29	1.15 ± 0.38	1.12 ± 0.41	1.17 ± 0.35	1.14 ± 0.38	<0.001
Non-HDL (mmol/L)	2.21 ± 1.06	2.01 ± 0.84	2.05 ± 0.93	2.1 ± 1.01	2.18 ± 0.99	2.14 ± 0.99	<0.001
Total cholesterol (mmol/L)	3.85 ± 1.76	4.12 ± 1.72	3.97 ± 1.78	3.93 ± 1.81	3.98 ± 1.78	3.95 ± 1.77	0.001
Total triglycerides (mmol/L)	1.9 ± 1.18	1.49 ± 0.88	1.79 ± 1.16	1.72 ± 1.06	1.69 ± 1.02	1.75 ± 1.1	<0.001
Hormone & renal function indicators							
Estradiol (pg/mL)	209.43 ± 110.57	182.1 ± 124.54	196.64 ± 112.9	196.66 ± 115.68	204.18 ± 117.93	200.59 ± 115.66	<0.001
Follicle-stimulating hormone (mIU/mL)	104.77 ± 53.43	93.76 ± 56.29	100.49 ± 56.01	103.79 ± 56.24	99.49 ± 55.23	101.25 ± 54.99	<0.001
Serum creatinine (*μ*mol/L)	103.44 ± 53.27	62.37 ± 28.96	87.64 ± 49.74	96.21 ± 59.22	87.71 ± 49.8	90.37 ± 51.57	<0.001
Uric acid (μmol/L)	263.4 ± 134.17	284.01 ± 90.29	273.93 ± 122.16	261.01 ± 129.02	272.5 ± 120.32	269.62 ± 122.75	<0.001
BMI (kg/m2)	24.13 ± 2.61	24.99 ± 2.76	24.31 ± 2.68	24.38 ± 2.69	24.59 ± 2.77	24.42 ± 2.7	<0.001
Tumor Markers							
CEA (ng/mL)	8.51 ± 18.47	9.35 ± 10.56	9.8 ± 23.87	9.49 ± 22.02	8.53 ± 11.89	8.93 ± 17.46	0.388
CA125 (U/mL)	64.31 ± 71.72	75.18 ± 56.2	73.95 ± 64.11	65.03 ± 54.32	71.23 ± 73.77	68.43 ± 66.43	<0.001
CA153 (U/mL)	29.8 ± 45.27	25.68 ± 40.45	31.27 ± 46.03	28.61 ± 43.08	28.47 ± 44.22	28.74 ± 43.97	0.073

### Prevalence and patterns of dyslipidemia

Overall, 2,838 patients (54.1%) had dyslipidemia at baseline (Table 2). Dyslipidemia prevalence differed across molecular subtypes (*p* < 0.001), highest in Luminal A (1,424/2,405; 59.2%) and lowest in Luminal B (HER2−) (504/1,148; 43.9%). Luminal B (HER2+) (254/467; 54.4%), HER2-enriched (341/620; 55.0%), and TNBC (315/606; 52.0%) showed intermediate-to-high prevalence.

**Table 2:** Prevalence and patterns of dyslipidemia at baseline by molecular subtype (*n* = 5,246).

N(%)	Total	Luminal A	Luminal B (HER2⁻)	Luminal B (HER2⁺)	HER2-enriched	Triple negative	*P* value
Dyslipidemia	2,838 (54.1%)	1,424 (59.2%)	504 (43.9%)	254 (54.4%)	341 (55.0%)	315 (52.0%)	<0.001
Elevated LDL-C	1,224 (23.3%)	758 (31.5%)	76 (6.6%)	103 (22.1%)	149 (24.0%)	138 (22.8%)	<0.001
Decreased HDL-C	1,019 (19.4%)	595 (24.7%)	103 (9.0%)	91 (19.5%)	135 (21.8%)	95 (15.7%)	<0.001
Elevated TC	1,550 (29.5%)	559 (23.2%)	393 (34.2%)	135 (28.9%)	165 (26.6%)	175 (28.9%)	<0.001
Elevated TG	1,349 (25.7%)	665 (27.7%)	263 (22.9%)	128 (27.4%)	140 (22.6%)	153 (25.2%)	0.009
Elevated TC & Elevated TG	342 (6.5%)	124 (5.2%)	100 (8.7%)	35 (7.5%)	42 (6.8%)	41 (6.8%)	0.002
Elevated TC & Elevated LDL-C	308 (5.9%)	184 (7.7%)	18 (1.6%)	25 (5.4%)	44 (7.1%)	37 (6.1%)	<0.001
Elevated TC & Decreased HDL-C	220 (4.2%)	120 (5.0%)	26 (2.3%)	19 (4.1%)	32 (5.2%)	23 (3.8%)	0.003
Elevated TG & Elevated LDL-C	63 (1.2%)	35 (1.5%)	5 (0.4%)	9 (1.9%)	5 (0.8%)	9 (1.5%)	0.036
Elevated TG & Decreased HDL-C	289 (5.5%)	148 (6.2%)	53 (4.6%)	34 (7.3%)	33 (5.3%)	21 (3.5%)	0.022
Elevated LDL-C & Decreased HDL-C	401 (7.6%)	240(10.0%)	28(2.4%)	40(8.6%)	50(8.1%)	43(7.1%)	<0.001

Across the cohort, elevated LDL-C was observed in 1,224 patients (23.3%), decreased HDL-C in 1,019 (19.4%), elevated TC in 1,550 (29.5%), and elevated TG in 1,349 (25.7%). Subtype differences were significant for elevated LDL-C, decreased HDL-C, and elevated TC (all *p* < 0.001), and also for elevated TG (*p* = 0.009). Several combined dyslipidemia patterns varied by subtype, including elevated TC with elevated TG (342/5,246; 6.5%; *p* = 0.002), elevated TC with elevated LDL-C (308/5,246; 5.9%; *p* < 0.001), elevated TC with decreased HDL-C (220/5,246; 4.2%; *p* = 0.003), elevated TG with elevated LDL-C (63/5,246; 1.2%; *p* = 0.036), elevated TG with decreased HDL-C (289/5,246; 5.5%; *p* = 0.022), and elevated LDL-C with decreased HDL-C (401/5,246; 7.6%; *p* < 0.001), indicating heterogeneous dyslipidemia phenotypes among molecular subtypes.

### Lipid control categories

When lipid values were categorized (Table 3), 2,526 patients (48.2%) had LDL-C < 2.6 mmol/L, whereas 990 (18.9%) had LDL-C ≥ 4.1 mmol/L; LDL-C category distributions differed significantly by subtype (*p* < 0.001). High LDL-C (≥4.1 mmol/L) was most frequent in Luminal A (619/2,405; 25.7%) and least frequent in Luminal B (HER2−) (58/1,148; 5.1%).Regarding HDL-C, 1,019 patients (19.4%) were classified as having low levels (<1.0 mmol/L), with subtype differences reaching statistical significance (*p* < 0.001). Low HDL-C was most commonly observed in Luminal A cases (595/2,405; 24.7%) and least common in Luminal B (HER2−) individuals (103/1,148; 9.0%).Most patients had TC < 5.2 mmol/L (3,823/5,246; 72.9%), while TG < 1.7 mmol/L was observed in 3,968 (75.6%), with significant subtype variation for both TC and TG category distributions (both *p* < 0.001).

**Table 3:** Baseline lipid control categories (LDL-C, HDL-C, TC, and TG) by molecular subtype.

Characteristic	Total	Luminal A	Luminal B (HER2⁻)	Luminal B (HER2⁺)	HER2-enriched	Triple negative	*P* value
LDL (mmol/L)							
Ideal level, LDL <2.6	2,526 (48.2%)	1,114 (46.3%)	590 (51.4%)	239 (51.2%)	299 (48.2%)	284 (46.9%)	<0.001
Appropriate level, 2.6 ≤ LDL < 3.4	1,506 (28.7%)	538 (22.4%)	483 (42.1%)	126 (27.0%)	173 (27.9%)	186 (30.7%)	
Borderline high, 3.4 ≤ LDL <4.1	224 (4.3%)	134 (5.6%)	17 (1.5%)	22 (4.7%)	21 (3.4%)	30 (5.0%)	
High LDL ≥4.1	990 (18.9%)	619 (25.7%)	58 (5.1%)	80 (17.1%)	127 (20.5%)	106 (17.5%)	
HDL (mmol/L)							<0.001
Low HDL, < 1.0	1,019 (19.4%)	595 (24.7%)	103 (9.0%)	91 (19.5%)	135 (21.8%)	95 (15.7%)	
Non-HDL (mmol/L)							<0.001
Ideal level, Non -HDL < 3.4	3,438 (65.5%)	1,375 (57.2%)	922 (80.3%)	326 (69.8%)	400 (64.5%)	415 (68.5%)	
Appropriate level, 3.4 ≤ Non -HDL < 4.1	1,034 (19.7%)	599 (24.9%)	109 (9.5%)	92 (19.7%)	136 (21.9%)	98 (16.2%)	
Borderline high, 4.1 ≤ Non -HDL < 4.9	729 (13.9%)	417 (17.3%)	101 (8.8%)	47 (10.1%)	78 (12.6%)	86 (14.2%)	
High level, Non -HDL ≥ 4.9	45 (0.9%)	14 (0.6%)	16 (1.4%)	2 (0.4%)	6 (1.0%)	7 (1.2%)	
TC (mmol/L)							
Appropriate level, TC < 5.2	3,823 (72.9%)	1,847 (76.8%)	755 (65.8%)	332 (71.1%)	456 (73.5%)	433 (71.5%)	<0.001
Borderline high, 5.2 ≤ TC <6.2	784 (14.9%)	258 (10.7%)	269 (23.4%)	82 (17.6%)	78 (12.6%)	97 (16.0%)	
High level, TC ≥ 6.2	639 (12.2%)	300 (12.5%)	124 (10.8%)	53 (11.3%)	86 (13.9%)	76 (12.5%)	
TG (mmol/L)							
Appropriate level, TG < 1.7	3,968 (75.6%)	1,779 (74.0%)	893 (77.8%)	345 (73.9%)	488 (78.7%)	463 (76.4%)	<0.001
Borderline high, 1.7 ≤ TG <2.3	350 (6.7%)	117 (4.9%)	127 (11.1%)	31 (6.6%)	29 (4.7%)	46 (7.6%)	
High level, TG ≥ 2.3	928(17.7%)	509(21.2%)	128(11.1%)	91(19.5%)	103(16.6%)	97(16.0%)	

### Factors associated with dyslipidemia

Subtype-stratified multivariable logistic regression models identified both shared and subtype-specific factors associated with dyslipidemia (Table 4). Age was significantly associated with dyslipidemia only in Luminal B (HER2−) (OR 1.027, 95% CI 1.013–1.042; *p* < 0.001), whereas the association was not significant in Luminal A (*p* = 0.098), Luminal B (HER2+) (*p* = 0.288), HER2-enriched (*p* = 0.877), and TNBC (*p* = 0.066).

**Table 4:** Subtype-stratified multivariable logistic regression for factors associated with dyslipidemia (ORs with 95% CIs).

Characteristic	Luminal A			Luminal B (HER2⁻)			Luminal B (HER2⁺)			HER2-enriched			Triple negative		
	OR	*P* value	95% C.I.	OR	*P* value	95% C.I.	OR	*P* value	95% C.I.	OR	*P* value	95% C.I.	OR	*P* value	95% C.I.
Age	1.009	0.098	0.998–1.02	1.027	<0.001	1.013–1.042	1.015	0.288	0.987–1.044	1.002	0.877	0.978–1.027	1.02	0.066	0.999–1.041
Hypertension	1.084	0.918	0.233–5.044	0.889	0.65	0.535–1.477	0.724	0.679	0.156–3.352	0.177	0.148	0.017–1.849	0.536	0.334	0.151–1.9
Diabetes mellitus	0.661	0.557	0.167–2.627	0.69	0.267	0.358–1.329	0.996	0.997	0.092–10.833	0.321	0.233	0.049–2.081	0.271	0.106	0.056–1.32
CHD	2.629	0.293	0.434–15.921	0.657	0.151	0.371–1.165	1.325	0.757	0.224–7.831	57.244	0.006	3.12–1,050.434	1.202	0.804	0.28–5.164
Electrocardiographic abnormalities															
Any arrhythmia	1.506	0.746	0.126–17.947	0.623	0.295	0.257–1.51	1.476	0.819	0.053–41.338	-	-	-	1.887	0.604	0.172–20.707
Tumor pathology & staging Indicators															
Lymph node metastasis	1.362	0.157	0.888–2.089	2.341	0.002	1.379–3.977	3.403	0.015	1.268–9.133	1.719	0.171	0.791–3.735	2.012	0.06	0.971–4.171
Intravascular tumor thrombus	1.841	<0.001	1.49–2.274	2.16	<0.001	1.595–2.924	4.02	<0.001	2.401–6.729	6.503	<0.001	3.238–13.061	9.795	<0.001	4.89–19.62
Clinical stage	0.682	0.044	0.47–0.99	1.131	0.53	0.77–1.662	0.865	0.713	0.398–1.879	0.557	0.082	0.288–1.076	0.979	0.944	0.548–1.75
Molecular pathology indicators															
E-cadherin	0.374	0.004	0.192–0.727	0.485	<0.001	0.35–0.671	0.901	0.8	0.403–2.016	1.552	0.396	0.562–4.289	0.91	0.757	0.499–1.657
TOPOII	1.237	0.062	0.99–1.546	0.886	0.441	0.65–1.207	1.034	0.922	0.534–2.003	1.121	0.843	0.363–3.463	1.217	0.506	0.683–2.171
P120 catenin	1.76	<0.001	1.355–2.285	0.977	0.897	0.687–1.39	1.042	0.888	0.583–1.863	1.409	0.52	0.496–4.002	0.832	0.411	0.537–1.289
CK5/6	0.918	0.613	0.659–1.279	1.316	0.536	0.551–3.142	1.255	0.534	0.613–2.571	1.058	0.931	0.297–3.763	0.806	0.329	0.522–1.243
Hormone & renal function indicators															
Estradiol (pg/mL)	1	0.785	0.999–1.001	1	0.391	0.998–1.001	0.999	0.387	0.997–1.001	1	0.961	0.998–1.002	0.999	0.52	0.998–1.001
Follicle-stimulating hormone (mIU/mL)	1	0.654	0.999–1.002	1.002	0.133	0.999–1.004	0.999	0.758	0.995–1.004	1	0.906	0.996–1.003	1.002	0.166	0.999–1.006
Serum creatinine (μmol/L)	1.001	0.114	1–1.003	1.002	0.438	0.997–1.006	0.999	0.643	0.994–1.004	1.003	0.029	1–1.007	0.998	0.284	0.994–1.002
Uric acid (μmol/L)	1	0.666	1–1.001	0.999	0.168	0.997–1	1	0.826	0.998–1.002	1.002	0.009	1–1.003	1.001	0.194	1–1.002
BMI (kg/m²)	1.02	0.285	0.984–1.057	1.053	0.069	0.996–1.113	1.096	0.078	0.99–1.214	0.961	0.339	0.885–1.043	0.933	0.081	0.864–1.009
Tumor markers															
CEA (ng/mL)	1.002	0.729	0.992–1.012	1.019	0.047	1–1.038	1.001	0.945	0.983–1.019	1.007	0.39	0.992–1.022	1.009	0.546	0.979–1.04
CA125 (U/mL)	1	0.778	0.998–1.001	0.996	0.001	0.993–0.998	0.993	0.002	0.989–0.998	1.002	0.214	0.999–1.006	0.998	0.235	0.995–1.001
CA153 (U/mL)	1	0.959	0.998–1.002	1.002	0.357	0.998–1.005	1.005	0.137	0.999–1.011	0.998	0.349	0.993–1.003	0.996	0.135	0.991–1.001

Tumor-related features showed consistent associations. Intravascular tumor thrombus showed a significant observational associated with dyslipidemia across all subtypes [Luminal A: OR: 1.841, *p* < 0.001; Luminal B (HER2−): OR: 2.160, *p* < 0.001; Luminal B (HER2+): OR: 4.020, *p* < 0.001; HER2-enriched: OR: 6.503, *p* < 0.001; TNBC: OR: 9.795, *p* < 0.001]. Lymph node metastasis was associated with dyslipidemia in Luminal B (HER2−) (OR 2.341; *p* = 0.002) and Luminal B (HER2+) (OR: 3.403; *p* = 0.015), but not in HER2-enriched (*p* = 0.171) or TNBC (*p* = 0.060).

Several additional markers demonstrated subtype-specific associations. In Luminal A, E-cadherin was inversely associated with dyslipidemia (OR: 0.374; *p* = 0.004), and P120 catenin was positively associated (OR: 1.760; *p* < 0.001); clinical stage also showed an inverse association (OR: 0.682; *p* = 0.044). In Luminal B (HER2−), E-cadherin remained inversely associated (OR: 0.485; *p* < 0.001), while CEA was positively associated (OR: 1.019; *p* = 0.047) and CA125 was inversely associated (OR: 0.996; *p* = 0.001). In Luminal B (HER2+), CA125 was inversely associated (OR: 0.993; *p* = 0.002). In HER2-enriched disease, CHD was associated with dyslipidemia (OR: 57.244; *p* = 0.006) with a very wide CI, and both serum creatinine (OR: 1.003; *p* = 0.029) and uric acid (OR: 1.002; *p* = 0.009) showed significant associations.

## Discussion

In this single-center cohort of treatment-naive patients with newly diagnosed breast cancer, dyslipidemia was common at baseline and differed substantially across molecular subtypes. Importantly, these findings reflect cardiovascular and lipid profiles at diagnosis before exposure to anticancer therapies that may further influence cardiometabolic risk. The overall prevalence of dyslipidemia (54.1%) indicates that lipid abnormalities are already present in more than half of patients at the time of diagnosis, supporting routine baseline cardiometabolic assessment as part of initial oncology work-up. Notably, we observed notable subtype heterogeneity: Luminal A exhibited the highest prevalence of dyslipidemia (59.2%), while Luminal B (HER2−) had the lowest (43.9%). Subtype differences were also observed for individual lipid abnormalities, including elevated LDL-C, decreased HDL-C, elevated TC, and elevated TG, suggesting that breast cancer patients do not share a uniform lipid phenotype at diagnosis, and subtype-informed evaluation may help refine early cardiovascular risk assessment.

Regression analyses identified observational associations between tumor-related features and dyslipidemia. Intravascular tumor thrombus demonstrated a consistent statistical correlation with dyslipidemia across all molecular subtypes, with the strongest association observed in TNBC. These observational findings should be interpreted with caution, as they may represent confounding by underlying tumor aggressiveness rather than direct causal or biological links. Lymph node metastasis was associated with dyslipidemia in Luminal B subtypes, suggesting that regional tumor spread may coincide with greater cardiometabolic burden in specific biological contexts. By contrast, age was independently associated with dyslipidemia only in Luminal B (HER2−) in this dataset, indicating that traditional host risk factors may not contribute uniformly across subtypes. Additionally, several exploratory subtype-specific biomarker associations were also observed, including inverse associations for E-cadherin in luminal disease and inverse associations for CA125 in Luminal B subtypes; these findings should be interpreted cautiously and primarily serve to generate hypotheses regarding tumor–metabolic interactions. In HER2-enriched disease, CHD showed a statistically significant association with dyslipidemia, but the extremely wide confidence interval suggests sparse-event bias and instability, so the magnitude of this association should not be over-interpreted without external validation and/or methods suited for rare-event modeling.

Overall, these observational findings support early lipid screening and cardiovascular risk assessment at the time of breast cancer diagnosis, with attention to subtype differences and tumor-related indicators such as intravascular tumor thrombus. Establishing baseline cardiometabolic profiles may also enable more accurate attribution of subsequent lipid changes to anticancer therapies and guide preventive interventions when indicated.

## Limitations

This study has limitations. First, the retrospective single-center design may introduce selection bias and limits generalizability. Because baseline characteristics were not systematically collected for excluded patients, we could not compare included and excluded populations to fully evaluate this bias. Second, residual confounding is possible because lifestyle factors (diet, physical activity, smoking), socioeconomic status, and prior lipid-lowering medication use were not comprehensively captured. Third, several cardiovascular outcomes and ECG abnormalities were rare, potentially leading to unstable regression estimates (e.g., very wide CIs for CHD in HER2-enriched disease). Fourth, no formal correction for multiple testing was performed, which may increase the risk of type I error; thus, all findings should be interpreted with appropriate caution. Finally, this analysis focused on baseline measurements; prospective longitudinal studies are needed to evaluate therapy-related cardiometabolic changes and subsequent cardiovascular outcomes.

## Conclusions

Dyslipidemia is common at baseline in treatment-naive patients with newly diagnosed breast cancer and varies across molecular subtypes. Intravascular tumor thrombus was consistently associated with dyslipidemia across all subtypes, and lymph node metastasis was associated with dyslipidemia in Luminal B subtypes. These findings support early lipid screening and cardiovascular risk assessment at diagnosis to optimize cardio-oncology care.

## Data Availability

The original contributions presented in the study are included in the article/Supplementary Material, further inquiries can be directed to the corresponding authors.
